# Prevalence and molecular characterization of Hepatitis B in HIV infected individuals in Botswana

**DOI:** 10.1186/1471-2334-14-S2-P86

**Published:** 2014-05-23

**Authors:** Motswedi Anderson, Simani Gaseitsiwe, Sikhulile Moyo, Terence Mohammed, Theresa K Sebunya, Jason Blackard, Joseph Makhema, Max Essex, Rosemary Musonda

**Affiliations:** 1 Botswana Harvard AIDS Institute Partnership, Gaborone, Botswana; 2 University of Botswana, Department of Biological Sciences, Gaborone, Botswana; 3 University of Cincinnati College of Medicine, Cincinnati, USA; 4 Harvard School of Public Health, Department of Immunology and Infectious Diseases, Boston, USA

## Introduction

Hepatitis B Virus (HBV) is a major coinfection in HIV infected patients and it has emerged as an important cause of morbidity and mortality in this group since the start of Highly Active Antiretroviral Therapy (HAART).HIV/HBV coinfection prevalence varies by geographic region even within the same country. In Botswana the coinfection prevalence data is very sparse and what is reported varies. Ten HBV genotypes with some subgenotypes have been described differing by geographic distribution, course of disease, response to treatment and development of mutations. Even though data have shown that genotypes are predictive of disease outcome, the circulating HBV genotypes in Botswana remains unknown. Therefore this study aims at determining the HIV\HBV coinfection prevalence rate and the circulating HBV genotypes in Botswana.

## Materials and methods

This is a retrospective cross sectional study of HIV individuals initiating HAART. Hepatitis B surface antigen (HBsAg) was screened for in 300 baseline samples and those which were positive were also screened for HBeAg. For the HbsAg positive a 415 bp fragment of the HBV polymerase gene was sequenced using Big Dye sequencing chemistry and the sequences were analyzed for genotypes and mutations.

## Results

Of the 300 participants, 28(9.3%) were HBsAg positive and 5(17.9%) were HBeAg positive. Of the 27 samples that were successfully genotyped, genotype A was found in 23 (85.2%), 22 subgenotype A1 and 1 subgenotypeA2, genotype D subgenotype D3 made up the remaining 4(14.8%). Known escape mutations were found in 12(44.4%) with the most common one being N131N found in 8(29.6%) of the participants. The other escape mutations were R122K, T123A, Q129R, G130N, M133T and F134V.No drug resistance mutations were detected.

## Conclusion

The HIV/HBV prevalence is consistent with what has been reported in the country. The predominant genotype in this cohort is A1 and its consistent with findings from the region. Escape mutations previously associated with failure of HBsAg detection and vaccine or immunogloblin therapy were found in a significant number of participants. No known drug resistance mutations were found in the cohort.

